# Supplementary figures and images for: Nitrogen and phosphorus losses by surface runoff and soil microbial communities in a paddy field with different irrigation and fertilization managements
Source: PLoS One. 2021 Jul 9;16(7):e0254227. doi: 10.1371/journal.pone.0254227 (PMC8274659; doi:10.1371/journal.pone.0254227)

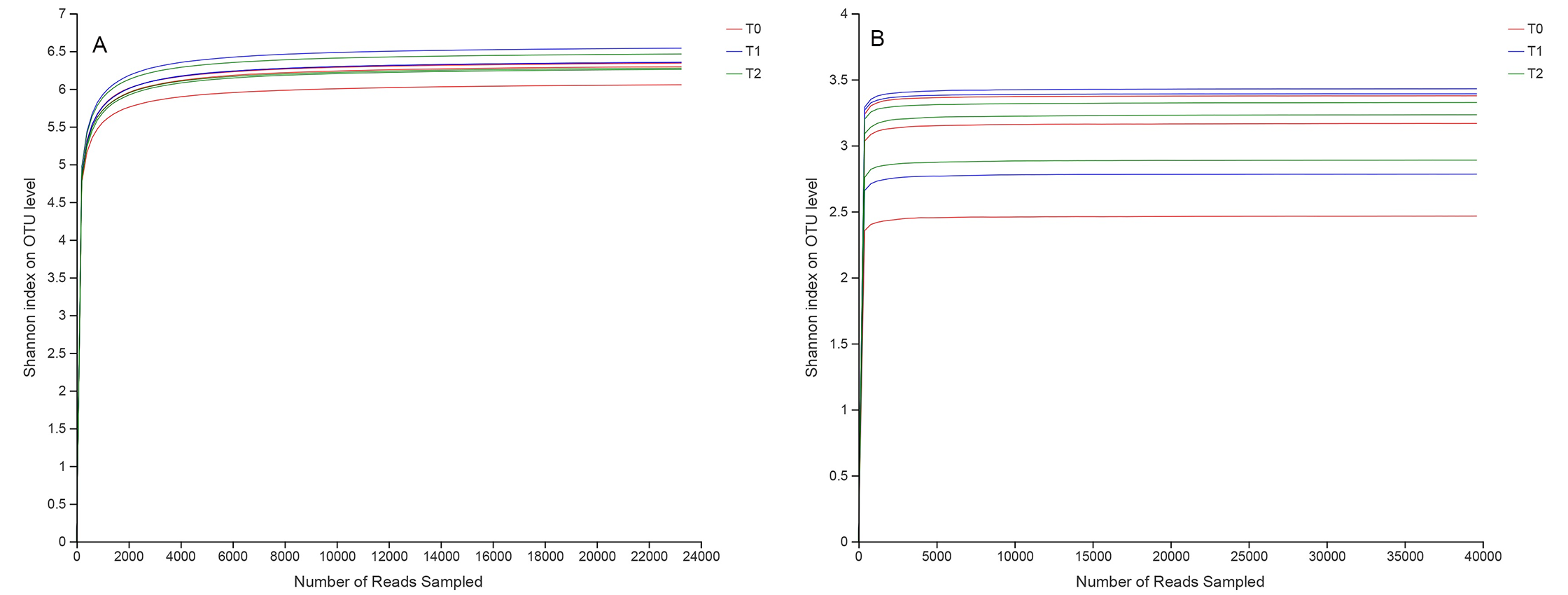

Supplement: S1 Fig — Bacterial (A) and fungal (B) Shannon–Wiener curves for normalized number of reads at a 97% threshold in different fertilization and irrigation regimes. Notes: T0 = Traditional irrigation; T1 = Traditional irrigation and fertilization practice; T2 = Water-saving irrigation and optimizing fertilization. (TIF) [file pone.0254227.s001.tif]
