# Supplementary material for: Nitrogen and phosphorus losses by surface runoff and soil microbial communities in a paddy field with different irrigation and fertilization managements
Source: PLoS One. 2021 Jul 9;16(7):e0254227. doi: 10.1371/journal.pone.0254227 (PMC8274659; doi:10.1371/journal.pone.0254227)
Supplement: Attachment — Submitted filename: Response to Reviewers.doc [file pone.0254227.s002.doc]

Reviewer #1:

The paper seems technically sound, but I have some doubts regarding the scientific validity and the rigor in science.

(1) Surface runoff should be primarily determined by water depth in the field and the size of rainfall events, rather than soil microorganism composition. In general, a higher water level results in more nutrient losses through surface runoff. Compared with the water depth (1-6 cm) in T_1_ (traditional irrigation), the water depth (-3-3 cm) in T_2_ (shallow intermittent irrigation) was relatively lower. Therefore, the risk of nutrient loss under T1 should be higher than it under T2. Hence, the major result of the decline of nutrient losses in T_2_ is not significant and surprised.

(2) There is a lack of figure, such as SEM, which comprehensively shows the direct effects of various environmental factors on nutrient losses, and its indirect effects through altering microorganism community composition. Besides studied environmental variables (Table 2), the size of rainfall events should be considered in investigating influencing factors of nutrient losses.

I have added the Fig 9A-B to comprehensively show the direct effects of various environmental factors on nutrient losses. In addition, runoff volume and fertilizer input were considered as the influencing factors of nutrient losses in Fig 9A-B.

Abstract

(1) The effect of microorganism community composition on nutrient runoff losses should be demonstrated specifically.

The effect of microorganism community composition on nutrient runoff losses was demonstrated specifically (lines 22-25 of revised manuscript with track changes).

(2) More details about T_1_ and T_2_ should be given.

More details about T_1_ and T_2_ have been given in lines 16-18, that is T_2_ (Chemical fertilizer of 240 kg N ha^−1^, 120 kg P ha^−1^, and 240 kg K ha^−1^ combined with shallow intermittent irrigation) and T_1_ (Chemical fertilizer of 273 kg N ha^−1^, 135 kg P ha^−1^, and 135 kg K ha^−1^ combined with traditional flooding irrigation).

Introduction

(1) Line 55-57, the relationship between ammonia oxidation and nitrogen losses should be elaborated.

Additionally, ammonia-oxidizing bacteria (AOB) played an important role in the ammonia oxidation which was crucial for N and P runoff losses ^[18]^. Wang et al. (2017) found that the ammonia oxidation contributed to 37.5–67.6% of N losses in the phreatic zone, where AOB might be the major source of nitrite nitrogen (NO_2_^-^–N) for ammonia-oxidizing bacteria ^[19]^ (lines 56-59).

(2) Line 81, the objective of this study was only to verify an existing irrigation practice. The “develop” is not appropriate.

The “develop” was deleted in line 82.

Materials and methods

(1) Line 107, why did you choose 20 cm as the height of the concrete cement border? I think the quantity of runoff is mainly depended on the height of the border.

That was because the height of the ridge is 20 cm.

(2) Line 109, it’s better to show a figure of the runoff collection device.

A figure of the runoff collection device was shown in Fig 1A-B.

Results

(1) Line 211, there are too many significant numbers.

Many significant numbers were deleted in line 229.

(2) The content of 3.5 part is not closely related to the main purpose of this study. The effects of abiotic factors on the microbial community composition should not be demonstrated in an independent part. The relationship between microorganisms and nutrient losses should be addressed specifically.

The content of 3.5 part is modified to “Factors impacting N and P surface runoff losses”, and thus rewrite this part. In addition, the relationship between microorganisms and nutrient losses should be addressed specifically (lines 294-306).

Discussion

(1) Line 285-286, please keep constant citation format in the text. Wang et a. (2019) is different from others.

Citation format was modified to constant citation format in the text (Lines 323-324 of revised manuscript with track changes).

(2) Line 285, the sentence “NO3--N was the main form of TN” doesn’t seem scientific.

The sentence was modified to “NO_3_^−^–N was the major form of N in the surface runoff” in line 325.

(3) Line 313, please delete one “the”.

One “the”was deleted in line 348.

(4) About 4.3 part, the relationship between microorganisms and environmental factors is not the critical issue in this study. Please rewrite and rename this part.

The content of 4.3 part was changed into “The influence of environmental factors on nitrogen and phosphorus losses”, and thus rewrite this part (lines 379-435).

Tables and Figures

(1) Only fertilization treatment was given in the Table 1. Please add irrigation treatment.

Fertilization and irrigation treatment was given in the Table 1 in lines 114-115.

(2) All tables and figures should be improved. The number and letters in the figures are too small.

All tables and figures were improved according to your journal requirements.

(3) About Fig. 2 B, Y-axis number “0.000-8.000” should be changed into “0-8”. Please correct other similar issues.

Too many decimal places In Fig. 2B and other similar issues were corrected.

(4) Figure resolution need to be improved.

Ensure that our images have a resolution of at least 300 pixels per inch (ppi) according to the full details of the requirements of figure preparation guidelines.

Reviewer #2: Dear Editor and authors,

PONE-D-20-38799 entitled “Water and fertilizer managements affect nitrogen and phosphorus losses by surface runoff and microbial communities in a paddy soil” have done nice work but being poorly presented. The MS maybe accepted after major revision as followings:

1. The language should be revised deeply through the MS, even in Title, please ask a specialist for help. The title should be “Nitrogen and phosphorus losses by surface runoff and soil microbial communities in a paddy field with different irrigation and fertilization managements”

The title and the language in the text were revised deeply through the MS withe track changes.

2. Line 14, please clearly define of T_1_.

More details about T_1_ and T_2_ have been given in lines 16-18, that is T_2_ (Chemical fertilizer of 240 kg N ha^−1^, 120 kg P ha^−1^, and 240 kg K ha^−1^ combined with shallow intermittent irrigation) and T_1_ (Chemical fertilizer of 273 kg N ha^−1^, 135 kg P ha^−1^, and 135 kg K ha^−1^ combined with traditional flooding irrigation).

3. Line 28 What is the optimum rice yield?

The “optimum” was deleted in line 29.

4. Line 36 the total cultivated area in where?

Rice (*Oryza sativa* L.) is one of the main staple crops and feeds over 65% of the world’s population with 11% of cultivated land ^[1-2]^ (lines 32-33).

5. In the introduction parts, the authors should clearly figure out the novelty of your study as there were plenty of literature on N and P losses from paddy fields with different water and fertilization managements.

To date, N and P runoff losses and their influencing factors while maintaining or enhancing rice yields in the paddy fields in southeastern China are currently unclear under different irrigation and fertilization regimes. Thus, we hypothesized that the appropriate irrigation and fertilization practices could affect N and P runoff losses by environmental factor variations.

6. Line 77 This knowledge gap is what? This should be clearly stated.

N and P runoff losses and their influencing factors while maintaining or enhancing rice yields in the paddy fields in southeastern China are currently unclear under different irrigation and fertilization regimes.

7. Table 1’ title should be Fertilization schemes for the different treatments. But the authors said that “The water and fertilizer practices used in this experiment are described in Table 1”, where is water managements? On other words, which kind of water-saving irrigation method you used in this study? Please clearly shown your experimental design, which is vital for the readers.

Fertilization and irrigation treatment was given in the Table 1 in lines 114-115.

8. 2.2 Water sampling methods should be described in details.

The detail description of water sampling methods is in lines 119-122.

9. Results this parts should be clearly and concise. Line 198 (2795 ± 165 t ha−1) should be deleted as the value has been shown in the Table 2, so many presentation in the results.

Many presentation, such as (2795 ± 165 t ha^−1^) in the results have been deleted.

10. Why you only shown the relate data obtained from 2018? If you want to make concise, please at least to state this in the Data analysis.

We wanted to make concise, and stated this in the Data analysis line 189.

11. Do not simply repeat the results in the Discussion parts.

The simply repeated results in the Discussion parts have been modified or deleted with track changes in the text.

12. The authors mentioned that “Runoff DN loss was mainly in the form of NO_3_-^–^N than in NH_4_^+^–N under different water and fertilizer treatments” and given the reasons. I thinks the differences in N and P losses among the different treatments was your mainly focus, and should be deeply discussed.

The reasons were given (lines 325-328). Moreover, the differences in N and P losses among the different treatments was deeply discussed (lines 348-356).

13. Is there any relationship between soil physiochemical properties and N and P losses? This should be one important novelty of this study.

The redundancy analysis (RDA) was conducted to determine which soil variables were related to N and P losses in Fig 9A-B, and described specially in lines 303-310 and 427-435.

14. The conclusions should be further clarified.

The conclusions were further clarified in lines 438-448.

15. All figures need to be clearly presented, and a lot of texts in figures are not visible.

Ensure that our images have a resolution of at least 300 pixels per inch (ppi) according to the full details of the requirements of figure preparation guidelines.
