# Supplementary material for: Nitrogen and phosphorus losses by surface runoff and soil microbial communities in a paddy field with different irrigation and fertilization managements
Source: PLoS One. 2021 Jul 9;16(7):e0254227. doi: 10.1371/journal.pone.0254227 (PMC8274659; doi:10.1371/journal.pone.0254227)
Supplement: Attachment — Submitted filename: Response to Reviewers.doc [file pone.0254227.s003.doc]

Additional Editor Comments (if provided):

I appreciate the revisions made. Although two reviewers agreed to accept the article, some additional comments are provided from an editorial standpoint. Most are rather specific and should be easy to address.

Specific comments:
Line 14. Change “Therefore” to “Here or In this study”.

“Therefore”was changed into “Here”in line 14 of revised manuscript with track changes.
Line 34. Please provide the full name of the abbreviation of N and P, though they have been defined in the abstract section.

We provided the full name of the abbreviation of N and P in line 33 of revised manuscript with track changes.
Line 36-37. Please cite one reference for this sentence.

We cited one reference for this sentence in line 37.
Line 37. Please add “,” between “To date” and “water-quality”.

We added “,” between “To date” and “water-quality”in line 37.
Line 49. Change “For example, Chen et al. (2018) reported that” to “It has been reported that”.

We Changed “For example, Chen et al. (2018) reported that” to “It has been reported that” in line 48.
Line 53. Change “For instance,” to “Related studies have suggested that”.

We Changed “For instance,” to “Related studies have suggested that”in line 51.
Line 57-60. Please consider the deletion of these sentences.

We deleted these sentences of lines 57-60.

Line 64-66. We all know that high-throughput sequencing is widely used in determining the diversity and composition of soil microbes. These sentences did not provide very useful information. Thus, please consider removing the sentences.

We removed the sentences of lines 64-66.

Line 67. Remove the “Moreover”.

We removed the “Moreover”in line 67.

Line 79-84. According to the title and abstract, different irrigation and fertilization practices could affect soil physicochemical properties and correspondingly influence soil microbial communities, and thereby contribute to N and P runoff losses. Am I right? However, this part has nothing to do with soil microorganisms. Your hypothesis also does not relate to microorganisms.

We Changed this part to “Thus, we hypothesized that different irrigation and fertilization practices could alter soil chemical properties and microbial community structure, which would subsequently affect N and P runoff losses. To test the hypothesis, a 10-year plot experiment was conducted to estimate N and P runoff losses and uptake, soil chemical properties, microbial diversity, and community composition under different fertilization and irrigation regimes. In general, the purpose of this study was to ⑴ verify an optimal irrigation and fertilization practice in order to minimize N and P runoff losses, and ⑵ explore the factors influencing N and P losses in surface runoff from paddy fields in southeastern China.” in lines 71-77.
Line 174-183. What did you do with singleton sequences (i.e., sequence appearing only one time in the entire data set)? Moreover, how do you address uneven sequencing depth across samples? Please be clearer in your presentation.

We revised this part according to editors' requirements in lines 169-181. Specifically, Using the UPARSE (version 7.0.1090), we also removed singleton sequences (i.e., sequences appearing only one time in the entire data set). As the number of sequence reads in each sample varied, the OTU table was rarified (holding the same sequence number in each sample) prior to microbial community diversity calculations.

Line 176-181. Please provide the versions of UPARSE, Silva rRNA database, Mothur software and insert references for them.

We provided the versions of UPARSE, Silva rRNA database, Mothur software and insert references for them in lines 171-179.
Line 179. The authors mentioned that you obtained rarefaction curves using Mothur software, where is the result of rarefaction curves?

We added the result of rarefaction curves in lines 251-253.
Line 193 and Line 200. Both Rstudio and R were used in this study to perform statistical analyses. Please provide their versions in your manuscript and insert reference.

We provided the versions of R in line 191 and line 199.
Line 194. Provide the version of the R pheatmap package and cite one reference.

We provided the versions of R in line 199.
Line 199-200. Which distance did you use? Please make it clear.

The unweighted UniFrac distance - based redundancy analysis (db‐RDA) was processed by R software (version 2.15.3) in lines 198-199.
Line 197. The “vif” is an abbreviation form. Please define it at its first mention.

We defined the “vif” as variance inflation factor in line 195.
Line 200. Provide the version of R vegan package, and then insert one reference for it.

We provided the versions of R in line 199, and then inserted one reference for it.
Line 241. Double “the”.

We deleted “the”in line 240.
Line 263-267. According to the description of microbial alpha diversity indices in Line 180, change “Ace” to “ACE” and change “Chao” to “Chao1” in the table 4. Meanwhile, provide the explanation of ACE in the table notes in Line 264-267.

We changed “Ace” to “ACE” and changed “Chao” to “Chao1” in the table 4. Meanwhile, provide the explanation of ACE (abundance-based coverage estimators) in the table notes in Line 265.

Line 306-307. Other environmental factor refer to what? And they would directly affect the N and P losses. I think this sentence can be removed as what is needed in the results section is for the author to describe their findings objectively.

This sentence “Other environmental factor refer to what? And they would directly affect the N and P losses” was removed in this manuscript.

Figures
Figure 1. This image needs to be cropped appropriately as there is a lot of white space throughout the image.

Figure 1 was cropped appropriately.
Figure 3. As an example, the font of the words “Runoff yield” and “TN loss loads” in the vertical titles did not seem to correspond to the font of the horizontal title. Please unify the font of all the text in the figure A, B, C and D. In addition, there are up ticks and right ticks in the X-axis and Y-axis of Fig. 3D, respectively, while all ticks in Fig. 3A, 3B and 3C are not shown. Please unify the drawing style. As for Fig. 3D, my suggestion is that the authors can show down ticks and left ticks in X-axis and Y-axis, respectively.

Figure 3 was revised according to editors' requirements.
Figure 3B. A point to note in the illustration of the types of line in the image is that the presentation of "NO_3_^-^-N" and "NH_4_^+^-N" should be revised as they are not presented in a very aesthetically pleasing way.

"NO_3_^-^-N" and "NH_4_^+^-N" in Figure 3 were revised.
Figure 3D. There are no data points between 0 and 0.6 mg/L of total phosphorus concentration, so the authors could have left the values of the horizontal coordinates not starting from 0.

Figure 3D was revised.
Figure 4. The word “Venn” and three solid dots with “T0/T1/T2” could be removed from your figure as they are redundant. In addition, the size of the words (e.g., T0, T1 and T2) and numbers in the Venn plots needs to be slightly adjusted upwards. Figure 4A: The bar diagram has two ticks at the value 200 of the vertical coordinate, please deal with them.

Figure 4 was improved according to editors' requirements.
Figure 5. Please delete the word “PLS-DA on OTU level” at the top of the figure.

The word “PLS-DA on OTU level” at the top of the figure 5 was deleted.
Figure 6. Only major phyla are presented in each treatment. What is the relative abundance of phyla below which they are classified as 'Others'? This needs to be made clear in the figure caption. In addition, please delete the word “Community barplot analysis” at the top of the figure.

Fig 6. Average relative abundance of dominant bacterial (A) and fungal (B) phyla (> 1.0%) in different fertilization and irrigation regimes. The abundance is expressed as the average percentage of the targeted sequences to the total high-quality bacterial and fungal sequences of samples from triplicate plots of each fertilization regime, respectively. Notes: ‘Others’ refer to those identified phyla with lower than 1.0% relative abundance in all the samples. T0 = Traditional irrigation; T1 = Traditional irrigation and fertilization practice; T2 = Water-saving irrigation and optimizing fertilization. In addition, we deleted the word “Community barplot analysis” at the top of the figure.
Figure 7. Please delete the word “db-RDA on OTU level” at the top of the figure.

We deleted the word “db-RDA on OTU level” at the top of the figure.
Figure 8. Please delete the word “Spearman Correlation Heatmap” at the top of the figure.

We deleted the word “Spearman Correlation Heatmap” at the top of the figure.
Figure 3-Figure 9: The numbers 0, 1 and 2 are below the letter T (i.e., the form of a subscript) in the figure captions and main text. However, in the figure, the author does not show the T0, T1 and T2 in a subscript form, so please standardize the format of presentation.

We standardized the format of presentation of T0, T1 and T2 in both Figure 3-Figure 9 and in this manuscript.
